# Virological suppression among gay, bisexual, and other men who have sex with men living with HIV in Vancouver, Canada: A longitudinal cohort study from 2012–2017

**DOI:** 10.1371/journal.pone.0276596

**Published:** 2022-10-21

**Authors:** Heather L. Armstrong, Julian Gitelman, Zishan Cui, Nicanor Bacani, Paul Sereda, Nathan J. Lachowsky, Kiffer G. Card, Jordan M. Sang, Henry F. Raymond, Julio Montaner, David Hall, Terry Howard, Mark Hull, Robert S. Hogg, Eric A. Roth, David M. Moore

**Affiliations:** 1 British Columbia Centre for Excellence in HIV/AIDS, Vancouver, Canada; 2 Department of Psychology, University of Southampton, Southampton, United Kingdom; 3 Faculty of Medicine, University of Toronto, Toronto, Canada; 4 School of Public Health and Social Policy, University of Victoria, Victoria, Canada; 5 School of Public Health, Rutgers University, Piscataway, New Jersey, United States of America; 6 Faculty of Medicine, University of British Columbia, Vancouver, Canada; 7 Immunodeficiency Clinic, St. Paul’s Hospital, Vancouver, Canada; 8 Momentum Health Study, Community Advisory Board, Vancouver, Canada; 9 Faculty of Health Sciences, Simon Fraser University, Burnaby, Canada; 10 Department of Anthropology, University of Victoria, Victoria, Canada; UNSW Australia, AUSTRALIA

## Abstract

**Introduction:**

In 2010, British Columbia (BC) implemented HIV Treatment as Prevention (TasP) as policy. We examined trends in virologic suppression and determinants of significant viremia among a prospective biobehavioural cohort of men who have sex with men (gbMSM) in Vancouver from 2012–2017.

**Methods:**

Respondent-driven sampling was used to recruit sexually active gbMSM (≥16 years) who completed biannual study visits with a computer-assisted self-interview and clinical CD4 and viral load (VL) testing. We linked participant data with the BC HIV Drug Treatment Program to obtain antiretroviral dispensing and VL data. We conducted a trend analysis of VL suppression using univariable generalized estimating equation (GEE) multi-level modelling and multivariable GEE to identify factors associated with episodes of VL ≥200 copies/mL.

**Results:**

Of 774 participants, 223 were living with HIV at baseline and 16 were diagnosed during follow-up (n = 239). We observed a significant trend towards reduced levels of unsuppressed VL (>200 copies/mL) from 22% (07/2012-12/2012) to 12% (07/2016-12/2016) (OR:0.87; 95%CI:0.83–0.91 for each 6-month period). Among those with at least one follow-up visit, (n = 178, median follow-up = 3.2 years, median age = 46.9 years), younger age (aOR:0.97; 95%CI:0.94–0.99, per year), ecstasy use (aOR:1.69; 95%CI:1.13–2.53), crystal methamphetamine use (aOR:1.71; 95%CI:1.18–2.48), seeking sex via websites (aOR:1.46; 95%CI:1.01–2.12), and lower HIV treatment optimism (aOR:0.94; 95%CI:0.90–0.97) were associated with episodes of elevated viremia.

**Conclusions:**

During a period when TasP policy was actively promoted, we observed a significant trend towards reduced levels of unsuppressed VL. Continued efforts should promote HIV treatment optimism and engagement, especially among younger gbMSM and those who use ecstasy and crystal methamphetamine.

## Introduction

In Canada, and in the province of British Columbia (BC), gay, bisexual, and other men who have sex with men (gbMSM) remain at disproportionately higher risk of contracting HIV. In 2017, 69.8% of all new HIV diagnoses in BC were among gbMSM [1]. HIV is highly endemic among gbMSM in Vancouver with a recent population prevalence estimate of 20.4% in 2017–2019 [2]. This study also found that 99.8% of gbMSM living with HIV in Vancouver were aware of their status, 88.5% were receiving antiretroviral therapy (ART), and 97.4% were virally suppressed (i.e., had a viral load under 200 copies/mL).

Transmission of HIV does not occur if viral levels remain below 200 copies/mL [3]. As such, HIV control policies have focused on efforts to expand HIV testing and to support engagement in HIV care and treatment in order to reduce viremia [4,5]. In BC, eligible residents living with HIV have been provided ART since 1992, with no co-payment or other costs to patients. Beginning in 2010, the province implemented Treatment as Prevention (TasP) as policy and expanded HIV testing, linkage, and engagement in care through a program known as Seek and Treat to Optimise Prevention of HIV (STOP/HIV; stophivaids.ca) [6].

The course of HIV infection is dynamic and it is common for patients with virologic suppression to experience viremic episodes [7,8]. Several factors including younger age [7,9–15] and belonging to an ethnic minority group [9,10,14–16] have been associated with less viral suppression in general, as well as lower treatment adherence and more episodes of elevated viremia; HIV treatment optimism has been previously associated with increased treatment adherence [17,18] which could lead to fewer episodes of unsuppressed VL. Other factors, such as stimulant and other drug use [7,9,11,14–16], participating in higher risk sexual behaviour [19–21], and mental health symptomology (including anxiety and depression) [14,16] have shown mixed results. Further, individuals who experience multiple overlapping syndemic factors (e.g., depressive symptoms and polysubstance use and condomless anal sex with casual partners) are more likely to experience episodes of elevated viremia compared to those who experience fewer syndemic factors [22]. A better understanding of how frequently gbMSM living with HIV experience episodes of unsuppressed viremia and factors associated with these periods could help inform strategies to improve the cascade of care, reduce viremia, and reduce onward transmission. Consequently, we examined trends in virologic suppression and the determinants of significant episodes of viremia among participants living with HIV in a longitudinal analysis of a prospective cohort of gbMSM in Metro Vancouver, Canada over a five-year period.

## Methods

We analyzed data from participants enrolled in the Momentum Health Study, a prospective biobehavioural cohort study of gbMSM in Metro Vancouver, British Columbia. Participants were recruited from February 2012 to February 2015 through respondent-driven sampling (RDS), a peer-recruitment strategy that uses purposefully chosen “seed” participants to target hidden and hard-to-reach populations [23]. Study visits occurred every six months; data up to February 2017 are included in this analysis. To be eligible, participants had to be 16 years of age or older, report having had sex with a man in the past 6 months, gender identify as men, live in Metro Vancouver, and be able to complete the study in English. Participants received $50 CAD for each study visit and an additional $10 CAD for each person they successfully recruited into the study. Full details of study methodology have been published elsewhere [24,25]. All study procedures were approved by the research ethics boards of the University of British Columbia, the University of Victoria, and Simon Fraser University. For the purpose of informed consent, the university ethics boards consider that individuals 16 years of age and older are capable of providing consent without parental approval.

Participants provided written informed consent before participating, including consent to link study data with data from the BC HIV Drug Treatment Program. For participants living with HIV in our cohort, this data linkage allowed us to access clinical patient data for VL and HIV treatment and enabled us to monitor those who missed study visits or were lost to follow-up, provided they remained within the province and engaged in care. All visits and study activities took place in the downtown study office, which was situated in the historically gay neighbourhood of Vancouver. At each visit, participants completed a computer-assisted self-interview (CASI) as well as a nurse-administered clinical survey. The CASI survey assessed socio-demographic, psychosocial, and behavioural variables, including sex and drug use. Relevant validated measures for the current analysis include the Hospital Anxiety and Depression Scale (HADS) [26] to assess mental health symptomatology and the HIV Treatment Optimism Skepticism Scale [27] for attitudes toward HIV treatment. During the nurse-administered clinical survey, participants who self-reported as HIV-positive at baseline had their serostatus confirmed by a point-of-care HIV test (Insti Rapid HIV-1/HIV-2 test; Biolytical Laboratories, Richmond, Canada) or through previous laboratory report obtained with their consent through their primary care provider or through electronic medical records. Viral load (VL) testing was performed each visit through St. Paul’s Hospital Laboratories if a recent result was not recorded in their electronic medical record. VL was measured using the Roche Amplicor Monitor assay (Roche Diagnostics, Laval, Canada) with lower limit of detection of the assay of 40 copies/ML. Participants who self-reported as HIV-negative at any study visit were administered a rapid HIV test and a 4^th^ generation enzyme immunoassay/antigen HIV test on a venous blood sample. The study sample for the present analysis includes all participants in the cohort who were diagnosed with HIV before or at enrolment and those who were diagnosed during the follow-up period.

We conducted a trend analysis using univariable generalized estimating equation (GEE) multi-level modelling (RDS chain: participant: visit), without RDS adjustments, with 6-month calendar time periods as the independent variable to determine the trend of viral load suppression over the course of the study. Study data and data linkage through the BC HIV Drug Treatment Program were used to classify participants into one of the following groups for each six-month study period: 1) newly diagnosed with HIV (unsuppressed), 2) receiving ART with VL <200 copies/mL (suppressed), 3) receiving ART with VL ≥200 copies/mL (unsuppressed), 4) receiving care (i.e., has had a VL or CD4 test in the last 6 months) but not receiving ART (unsuppressed), 5) previously diagnosed but not in care, as evidenced by no record of CD4 or VL result in a 6-month period (unsuppressed), 6) lost to follow-up (excluded), and 7) deceased or known to have moved out of province (excluded). Any episode of elevated viremia during a 6-month period was considered as “unsuppressed”.

Among participants with at least one follow-up visit, univariable and multivariable GEE multi-level modelling (RDS chain: participant: visit), without RDS adjustments, was used to identify factors associated with any episode of VL ≥200 copies/mL during a 6-month period. Demographic variables of interest included age, ethnicity, neighbourhood of residence, annual income, and sexual identity. Additional variables tested over the last six months included: drug use (including ecstasy, ketamine, gamma hydroxy-butyrate [GHB], crystal methamphetamine, and injection drug use not including steroid use); number of anal sex partners; any condomless anal sex with known HIV-negative, HIV-positive, and unknown serostatus partners; any transactional sex; any attendance at gay bars/clubs; any Internet website or mobile app use to meet sexual partners; frequency of asking about sexual partners’ HIV statuses; and frequency of telling one’s own HIV status to sexual partners. Scores on the HADS and the HIV Treatment Optimism Skepticism Scale were also considered for the model. All variables with *p*<0.20 in univariable analyses were considered for inclusion in the final multivariable model. Model selection was conducted using a backward selection technique to eliminate the variable with largest Type III *p*-value until QIC was minimized [28]. All analyses were conducted using SAS Version 9.4 (SAS Corporation, Cary NC).

## Results

**Fig 1:**
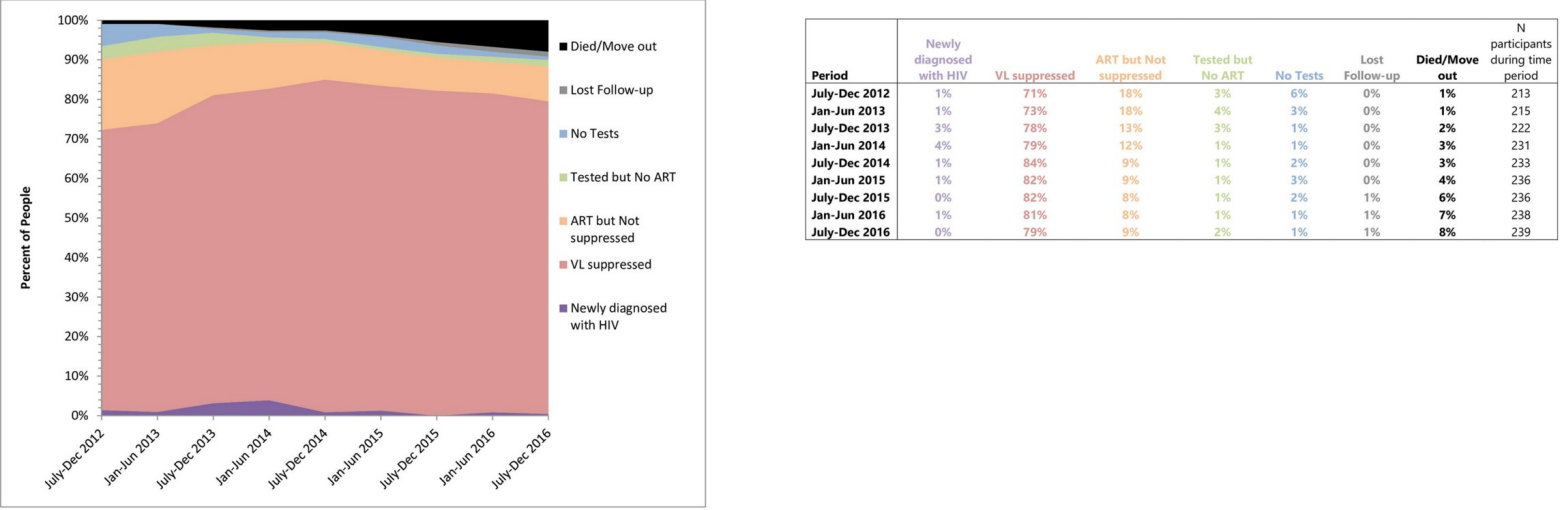
Cascade of care for gbMSM living with HIV in the Momentum Health Study 2012–2017.

We enrolled 774 participants, of which 134 (17.3%) were initial seeds. At their first study visit, 223 were living with HIV and an additional 16 participants were diagnosed with HIV during the follow-up period (n = 239 total). In the first six-month period (July 2012-December 2012), 71% had VL suppression, 18% were on ART without VL suppression, 6% had been previously diagnosed but were not in care, 3% had been previously diagnosed and were in care but were not on ART, and 1% were newly diagnosed with HIV; 1% were known to have moved out of province or had died. In the last six-month period (July 2016-December 2016), 79% had VL suppression, 9% were on ART without VL suppression, 1% had been previously diagnosed but were not in care, 2% had been previously diagnosed and were in care but were not on ART, and 0% were newly diagnosed with HIV; 1% were lost to follow-up and 8% were known to have moved out of province or had died (Fig 1). After excluding those who were lost to follow-up or who had moved out of province or died, the proportion of participants with unsuppressed VL (≥200 copies/mL) significantly decreased over the course of the study from 22% of participants in the first six-month period (July 2012-December 2012) to 12% in the final period (July 2016-December 2016) (OR: 0.87; 95% CI: 0.83–0.91 for each 6-month period; *p*<0.0001 for trend). On average, 15.2% of the sample had an unsuppressed VL in each six-month period with an additional average of 2.2% without any CD4 or VL testing. Over the course of the study, 3 individuals living with HIV were lost to follow-up from the study and an additional 6 died and 13 moved out of province.

Of the 239 participants living with HIV, 178 had at least one follow-up study visit and were included in the analysis examining factors associated with having an unsuppressed VL. These individuals contributed a total of 1284 study visits in a median of 3.2 years (95%CI: 2.9–4.1) of follow-up. The median age at baseline was 46.9 years (95% CI: 39.1, 51.7), most identified as gay (86.0%), were White (80.3%), lived in downtown Vancouver (64.6%), and had an annual income less than $30,000 CAD (69.1%). Please see Table 1 for full statistics.

**Table 1:** Descriptive statistics of sample of gbMSM living with HIV included in GEE modelling (n = 178).

	Overall (n = 178)		VL Suppressed (n = 127)		VL Unsuppressed (n = 51)		*p*-value
	Median	Q1, Q3	Median	Q1, Q3	Median	Q1, Q3	
**Age**	46.9	39.1, 51.7	47.7	41.8, 52.8	42.6	30.9, 50.0	**0.002**
**HADS Depression Scale**	4	2, 7	4	2, 6	3.5	2, 8	0.86
**HADS Anxiety Scale**	7	4, 11	8	4, 11	6.5	4, 11	0.45
**HIV Treatment Optimism Skepticism Scale**	29	26, 32	29	26, 32	28	24, 32	0.55
**P6M # of male sex partners**	8	3, 20	8	2, 20	8	4, 32	0.18
**P6M # of male anal sex partners**	5	2, 20	5	1, 20	5	3, 22	0.12
	n	%	n	%	n	%	
**Sexual Identity**							
Gay	153	86.0	108	85.0	45	88.2	0.64
Bisexual/Other	25	14.0	19	15.0	6	11.8	
**Ethnicity**							
White	143	80.3	106	83.5	37	72.6	0.08
Asian/Latino/Other	20	11.2	14	11.0	6	11.8	
Indigenous	15	8.4	7	5.5	8	15.7	
**Annual Income**							
<$30,000	123	69.1	89	70.1	34	66.7	0.57
$30,000-$59.999	40	22.5	29	22.8	11	21.6	
≥$60.000	15	8.4	9	7.1	6	11.8	
**Neighbourhood**							
Downtown	115	64.6	87	68.5	28	54.9	0.22
Elsewhere Vancouver	39	21.9	25	19.7	14	27.5	
Outside Vancouver	24	13.5	15	11.8	9	17.7	
**Received any Money/Drugs/Goods for Sex (P6M)**	37	20.8	22	17.3	15	29.4	0.10
**Ecstasy Use (P6M)**	46	25.8	26	20.5	20	39.2	**0.01**
**Ketamine Use (P6M)**	35	19.7	20	15.8	15	29.5	0.06
**GHB Use (P6M)**	63	35.4	40	31.5	23	45.1	0.12
**Crystal Meth Use (P6M)**	82	46.1	52	40.9	30	58.8	**0.045**
**IDU Use not including Steroids (P6M)**	23	12.9	15	11.8	8	14.7	0.47
**Seeking Sex via Websites (P6M)**	127	71.4	88	69.3	39	58.8	**0.03**
**CAS with HIV-neg Partner (P6M)**	54	30.5	34	27.0	20	39.2	0.15
**Frequency of Asking Partner’s HIV status**							
<50% of the time	78	43.8	60	47.2	18	35.3	0.18
≥50% of the time	100	56.2	67	52.8	33	64.7	
**Frequency of Telling Partners own Status**							
<50% of the time	35	19.7	27	21.3	8	15.7	0.34
≥50% of the time	121	68.0	87	68.5	34	66.7	
Only when Asked	22	12.4	13	10.3	9	17.7	

Factors associated with any instance of unsuppressed VL (≥200 copies/mL) in a 6-month calendar period, analysed with univariable and multivariable GEE, are presented in Table 2. In multivariable analysis, periods of elevated viremia were independently associated with younger age (adjusted OR [aOR] = 0.97, 95% CI: 0.95–0.99, per year), using websites to find sexual partners during the past 6 months (aOR = 1.46, 95% CI: 1.01–2.12), ecstasy (aOR = 1.69, 95% CI: 1.13–2.53) and crystal methamphetamine use during the past 6 months (aOR = 1.71, 95% CI: 1.18–2.48), and less HIV treatment optimism (aOR = 0.94, 95% CI: 0.90–0.97). Despite being significantly associated with unsuppressed VL in univariable analyses, Indigenous ethnicity (vs White), reported receipt of money, drugs, or goods in exchange for sex, depressive symptoms, and condomless anal sex (CAS) with an HIV-negative partner were no longer associated with VL after controlling for other variables in multivariable model.

**Table 2:** Factors associated with episodes of VL ≥ 200 copies/mL over time.

	Univariable GEE				Multivariable GEE			
	OR	95% CI		p-value	AOR	95% CI		p-value
**Age (period based)**	0.97	0.94	0.99	0.003*	0.97	0.95	0.999	0.044*
**HIV Treatment Optimism Skepticism Scale**	0.93	0.90	0.97	0.000*	0.94	0.90	0.97	0.001*
**P6M # of anal sex partners**	1.00	1.00	1.00	0.327				
**Sexual Identity**								
Gay	1.00							
Bisexual/Other	0.94	0.41	2.15	0.882				
**Ethnicity**								
White	1.00				0.53	0.19	1.51	0.237
Asian/Latino/Other	0.55	0.28	1.06	0.072	0.32	0.10	1.00	0.050
Indigenous	2.72	1.13	6.56	0.026*	1.00			
**Annual Income**								
<$30,000	1.00							
$30,000-$59.999	1.03	0.65	1.65	0.895				
≥$60.000	0.94	0.49	1.82	0.862				
**P6M Received Any Money/Drugs/Goods for Sex**								
No	1.00				Not selected			
Yes	1.66	1.01	2.74	0.047*				
**P6M Ecstasy**								
No	1.00				1.00			
Yes	1.88	1.28	2.75	0.001*	1.69	1.13	2.53	0.010*
**P6M Ketamine**								
No	1.00							
Yes	1.30	0.82	2.05	0.265				
**P6M GHB**								
No	1.00							
Yes	1.23	0.83	1.82	0.307				
**P6M Crystal Meth**								
No	1.00				1.00			
Yes	1.91	1.32	2.74	0.001*	1.71	1.18	2.48	0.005*
**P6M Intravenous Drug Use (not including steroids)**								
No	1.00				Not selected			
Yes	1.46	0.93	2.29	0.103				
**P6M Seeking Sex via Websites**								
No	1.00				1.00			
Yes	1.63	1.14	2.34	0.008*	1.46	1.01	2.12	0.046*
**P6M Condomless Anal Sex HIV-negative Partner**								
No	1.00				1.00			
Yes	0.68	0.47	0.99	0.043*	0.71	0.49	1.03	0.074
**Ask Status Frequency**								
< 50% of the time	1.00							
≥ 50% of the time	1.14	0.81	1.59	0.456				
**Tell Status Frequency**								
< 50% of the time	1.00							
≥ 50% of the time	1.38	0.91	2.10	0.132				
Only when asked	1.27	0.80	2.01	0.319				
**HADS Depression**								
None	1.00				Not selected			
Mild/Moderate/Severe	1.42	1.02	1.97	0.039*				
**HADS Anxiety**								
None	1.00							
Mild/Moderate/Severe	1.02	0.67	1.55	0.941				

Note: Variables "not selected" were tested for inclusion in the multivariable model but were not retained in the final model due to model fit considerations.

## Discussion

Among a community-based sample of gbMSM recruited through respondent-driven sampling, our results demonstrate a significant trend towards reduced levels of unsuppressed VL among gbMSM living with HIV in Vancouver during a period when TasP was actively promoted as policy in BC. After excluding participants lost to follow-up and those who had moved out of province or had died, we found that 91% of participants living with HIV in our study were receiving ART and 72% were virologically suppressed in the first study period (July-December 2012); this increased to 97% of participants receiving ART and 87% having a suppressed VL during the last study period (July-December 2016). Our results are consistent with a previous analysis of all MSM living with HIV in BC identified in the Drug Treatment Program which found that by 2015, 89% were receiving ART and 80% were virologically suppressed [29]. Further, these results suggest that efforts to promote Treatment as Prevention have been largely successful and that among gbMSM in Vancouver, the new UNAIDS 2030 targets of 95% of people living with HIV diagnosed, 95% of those diagnosed on treatment, and 95% of those on treatment with viral suppression [30] have already been exceeded, as under 95-95-95, 85.7% of the population of people living with HIV would have a suppressed VL and we observed a level of 87%.

However, even with strong support and resources to support the TasP policy, a small minority of individuals experience episodes of elevated viremia over time and are potentially able to transmit the virus to their sexual partners. Among participants with at least two study visits, we found that episodes of unsuppressed viral load were associated with younger age (per year), using websites to meet sex partners, using ecstasy and crystal methamphetamine, and reporting less HIV treatment optimism. Previous research has likewise found that younger age is associated with failure to achieve viral suppression [7,9–12] and likelihood of experiencing any episode of viral rebound after viral suppression [7,10], potentially due to difficulties with retention in care [13]. In a large review of determinants of HIV incidence disparities among younger and older gbMSM in the United States, younger gbMSM living with HIV were more likely than older gbMSM to experience depression, poly-substance use, low income, and decreased health care access, as well as to engage in high risk sexual behaviour [12], factors which may contribute to reduced treatment adherence, elevated viremia, and onward transmission. As such, younger gbMSM remain a key demographic and additional efforts should be made to retain them in care to increase rates of viral suppression and decrease periods of elevated viremia, ultimately leading to better individual clinical outcomes and reduced community spread of HIV.

Even after controlling for effects of age in this analysis, recent (past six month) stimulant use (i.e., ecstasy and/or crystal methamphetamine) was associated with episodes of elevated viremia. Stimulant use has been previously associated with nonadherence to antiretroviral treatment, unsuppressed viral load, and viral rebound [31,32]. This is concerning as crystal methamphetamine and ecstasy are often used in sexualised contexts (e.g., party and play (PnP), chemsex) [33] and crystal methamphetamine in particular has been associated with greater likelihood of engaging in higher-risk sexual behaviours and STI diagnosis [34–36]. In a recent analysis of crystal methamphetamine use among gbMSM living with HIV in the Momentum cohort, 44.3% reported any crystal methamphetamine use at enrolment and this was stable across time; most who used reported using at least monthly [37]. Further, crystal methamphetamine use was associated with more recent anal sex partners, more condomless sex with seroconcordant partners, trading sex for drugs, recent STI diagnosis, and greater use of poppers, GHB, and ecstasy [37]. As individuals who use stimulants are more likely to experience episodes of elevated viremia and are more likely to participate in higher risk sexual behaviour, there is an increased likelihood that onward transmission may occur. As individuals increasingly shift to biomedical HIV prevention strategies such as TasP and U = U, targeted interventions promoting condom use among gbMSM living with HIV who use stimulants are still needed.

Finally, participants who scored higher on the HIV Treatment Optimism Skepticism Scale were less likely to have episodes of unsuppressed viral load. These findings are likely bidirectional as those who feel more optimistic about their medications may be more likely to adhere to their treatment program [17,18] which in turn is likely to improve overall health and well-being, leading to greater optimism and continued adherence. Therefore, continuing to highlight the benefits of treatment adherence for self, partners, and community may further help to reduce episodes of elevated viremia among individuals, leading to fewer potential opportunities for transmission within the community.

The results of this study need to be considered within the context of several limitations. First, while RDS aims to recruit a more representative sample of gbMSM from the larger population and produce population parameter estimates, it is unknown how our sample reflects the general gbMSM population in Metro Vancouver. However, this sampling approach addresses limitations of clinic-based recruitment or administrative data analysis as it includes men who were not engaged in care and those who eventually seroconverted. Second, given the strong provincial support for HIV testing, TasP, and retention in care for individuals living with HIV, our results may not be generalizable to gbMSM or other populations in other locations with fewer supports and resources. Third, our results may be susceptible to cohort effects. Specifically, as people age and the longer they live with HIV, they may become more adherent to medication and those who are more adherent to ART and who have more stable living conditions may be more likely to remain in the study over time. For our VL trend analysis, this concern is mitigated by our ability to link our sample to the provincial Drug Treatment Program which enabled us to access additional data on VL and HIV treatment and increased our ability to follow our sample over time, thereby reducing the effects of loss-to-follow up typical of observational cohorts; however, this may have affected our ability to detect factors associated with episodes of elevated viremia in GEE modelling. Fourth, frequency of unsuppressed VL was fairly low in this sample and as a result, this may have limited our statistical power to identify weaker associations. Additionally, our study itself may be considered as a type of intervention as participants in our study were linked to care as part of their study participation. Fifth, in our GEE models, the median age of our sample at first study visit was 46.9 years and there were few participants under 30 years. While this reflects decreasing trends of new HIV diagnosis within the province, it limits our ability to explore age effects among our sample. Finally, our data were collected before the beginning of the COVID-19 pandemic and as such may not be reflective of any changes in episodes of viremia during this time.

## Conclusions

Results of this study provide encouraging support that, at least in some contexts and with strong community, environmental, and political support and resourcing, increasing treatment adherence and reducing periods of unsuppressed viral load is feasible [1]. However, even with strong support and resources, some gbMSM continue to experience episodes of elevated viremia, particularly younger gbMSM and those who use stimulants, including ecstasy and crystal methamphetamine. Given that higher rates of HIV treatment optimism were associated with fewer episodes of viremia, continued efforts promoting engagement and highlighting the benefits of treatment adherence among all gbMSM may further decrease episodes of elevated of viremia among gbMSM living with HIV and ultimately transmission rates.

## References

[1] BC Centre for Disease Control. HIV in British Columbia: Annual surveillance report 2017. 2019. Available at: http://www.bccdc.ca/health-professionals/data-reports/hiv-aids-reports.

[2] Moore DM, Cui Z, Skakoon-Sparling S, Sang J, Barath J, Wang L, et al. Characterisitics of the HIV cascade of care and unsuppressed viral load among gay, bisexual and other men who have sex with men living with HIV across Canada’s three largest cities. J Int AIDS Soc. 2021;24:e25699. 33929091 10.1002/jia2.25699 PMC8086033

[3] Rodger AJ, Cambiano V, Bruun T, Vernazza P, Collins S, Van Luzen J, et al. Sexual activity without condoms and risk of HIV transmission in serodifferent couples when the HIV-positive partner is using suppressive antiretroviral therapy. JAMA. 2016;316:171. doi: 10.1001/jama.2016.5148 27404185

[4] Centers for Disease Control and Prevention, Health Resources and Services Administration, National Institutes of Health, American Academy of HIV Medicine, Association of Nurses in AIDS Care, International Association of Providers of AIDS Care, the National Minority AIDS Council, and Urban Coalition for HIV/AIDS Prevention Services. Recommendations for HIV prevention with adults and adolescents with HIV in the United States, 2014. Available at: http://stacks.cdc.gov/view/cdc/26062.

[5] UNAIDS. 90-90-90: An ambitious treatment target to help end the AIDS epidemic. Geneva: UNAIDS, 2014 Available at: http://www.unaids.org/sites/default/files/media_asset/90-90-90_en_0.pdf.

[6] BC-CfE | STOP HIV/AIDS. Available at http://stophivaids.ca.

[7] Tanner Z, Lachowsky N, Ding E, Samji H, Hull M, Cescon A, et al. Predictors of viral suppression and rebound among HIV-positive men who have sex with men in a large multi-site Canadian cohort. BMC Infect Dis. 2016;16:1–11. 27769246 10.1186/s12879-016-1926-z PMC5073906

[8] Myer L, Dunning L, Lesosky M, Hsiao NY, Phillips T, Petro G, et al. Frequency of viremic episodes in HIV-infected women initiating antiretroviral therapy during pregnancy: A cohort study. Clin Infect Dis. 2017;64:422–427. doi: 10.1093/cid/ciw792 27927852 PMC5849096

[9] Yehia BR, French B, Fleishman JA, Metlay JP, Berry SA, Korthuis PT, et al. Retention in care is more strongly associated with viral suppression in HIV-infected patients with lower versus higher CD4 counts. J Acquir Immune Defic Syndr. 2014;65:333–339. doi: 10.1097/QAI.0000000000000023 24129370 PMC3945404

[10] Cohen SM, Hu X, Sweeney P, Johnson AS, Hall HI. HIV viral suppression among persons with varying levels of engagement in HIV medical care, 19 US jurisdictions. J Acquir Immune Defic Syndr. 2014;67:519–527. doi: 10.1097/QAI.0000000000000349 25230292 PMC11966502

[11] Palmer A, Gabler K, Rachlis B, Ding E, Chia J, Bacani N, et al. Viral suppression and viral rebound among young adults living with HIV in Canada. Medicine. 2018; 97. doi: 10.1097/MD.0000000000010562 29851775 PMC6392935

[12] Jeffries WL, Greene KM, Paz-Bailey G, McCree DH, Scales L, Dunville R, et al. Determinants of HIV incidence disparities among young and older men who have sex with men in the United States. AIDS Behav. 2018;22:2199–2213. doi: 10.1007/s10461-018-2088-3 29633094

[13] Yehia BR, Rebeiro P, Althoff KN, Agwu AL, Horberg MA, Samji H, et al. The impact of age on retention in care and viral suppression. J Acquir Immune Defic Synd. 2015;68:413–419. 10.1097/QAI.0000000000000489 PMC4334738 25559604

[14] Craw JA, Beer L, Tie Y, Jaenicke T, Shouse RL, Prejean J. Viral rebound among persons with diagnosed HIV who achieved vrial suppression, United States. J Acquir Immune Defic Synd. 2020;84:133–140. 10.1097/QAI.0000000000002321 PMC7604780 32084054

[15] Robertson M, Laraque F, Mavronicolas H, Braunstein S, Torian L. Linkage and retention in care and the time to HIV viral suppression and viral rebound—New York City. AIDS Care. 2015;27:260–267. doi: 10.1080/09540121.2014.959463 25244545

[16] Crawford TN. Poor retention in care one-year after viral suppresion: A significant predictor of viral rebound. AIDS Care. 2014;26:1393–1399. 24848440 10.1080/09540121.2014.920076

[17] Holmes WC, Pace JL. HIV-seropositive individuals’ optimistic beliefs about prognosis and relation to medication and safe sex adherence. J Gen Intern Med. 2002;17:677–68. doi: 10.1046/j.1525-1497.2002.00746.x 12220363 PMC1495106

[18] Godin G, Côté J, Naccache H, Lambert LD, Trottier S. Prediction of adherence to antiretroviral therapy: A one-year longitudinal study. AIDS Care. 2005;17:493–504. doi: 10.1080/09540120412331291715 16036235

[19] Mattson CL, Freedman M, Fagan JL, Frazier EL, Beer L, Huang P, et al. Sexual risk behavior and viral suppresion among HIV-infected adults receiving medical care in the United States. AIDS. 2014;15:1203. 10.1097/QAD.0000000000000273 PMC4004641 25000558

[20] Wilson PA, Kahana SY, Fernandez MI, Harper GW, Mayer K, Wilson CM, et al. Sexual risk behavior among virologically detectable human immunodeficiency virus-infected young men who have sex with men. JAMA Pediatrics. 2016;170:125–131. doi: 10.1001/jamapediatrics.2015.3333 26641367 PMC4821589

[21] Huerga H, Venables E, Ben-Farhat J, van Cutsem G, Ellman T, Kenyon C. Higher risk sexual beahviour is associated with unawareness of HIV-positivity and lack of viral suppression: Implications for Treatment as Prevention. Sci Rep. 2017;7:1–7. 29170407 10.1038/s41598-017-16382-6 PMC5700952

[22] Friedman MR, Stall R, Plankey M, Chongyi WE, Shoptaw S, Herrick A, et al. Effects of syndemics on HIV viral load and medication adherence in the Multicenter AIDS Cohort Study. AIDS. 2015;29:1087. 25870981 10.1097/QAD.0000000000000657 PMC4739626

[23] Lachowsky NJ, Sorge JT, Raymond HF, Cui Z, Sereda P, Rich A, et al. Does size really matter? A sensitivity analysis of number of seeds in a respondent-driven sampling study of gay, bisexual and other men who have sex with men in Vancouver, Canada. BMC Med Res Methodol. 2016;16:1–10. 27852234 10.1186/s12874-016-0258-4 PMC5112687

[24] Moore DM, Cui Z, Lachowsky N, Raymond HF, Roth E, Rich A, et al. HIV community viral load and factors associated with elevated viremia among a community-based sample of men who have sex with men in Vancouver, Canada. J Acquir Immune Defic Syndr. 2016;72:87–95. doi: 10.1097/QAI.0000000000000934 26825177 PMC4837069

[25] Forrest JI, Lachowsky NJ, Lal A, Cui Z, Sereda P, Raymond HF, et al. Factors associated with productive recruiting in a respondent-driven sample of men who have sex with men in Vancouver, Canada. J Urban Health. 2016;93:379–387. doi: 10.1007/s11524-016-0032-2 26960428 PMC4835350

[26] Zigmond AS, Snaith RP. The hospital anxiety and depression scale. Acta Psychiatr Scand. 1983;67:361–370. doi: 10.1111/j.1600-0447.1983.tb09716.x 6880820

[27] Ven PVD, Crawford J, Kippax S, Knox S, Prestage G. A scale of optimism-scepticism in the context of HIV treatments. AIDS Care. 2000;12:171–176. doi: 10.1080/09540120050001841 10827857

[28] Lima VD, Geller J, Bansberg DR, Patterson TL, Daniel M, Kerr T, et al. The effect of adherence on the association between depressive symptoms and mortality among HIV-infected individuals first initiating HAART. AIDS. 2007;21:1175–1183. doi: 10.1097/QAD.0b013e32811ebf57 17502728

[29] Moore DM, Ye M, Wong J, Barrios R, Hull M, Li J, et al. Unsuppressed viral load (VL) by HIV exposure category among people living with HIV in British Columbia (BC) Canada: 2005–2015. Vancouver, Canada, 2019.

[30] UNAIDS Joint United Nations Programme on HIV/AIDS. Understanding Fast-track: Accelerating action to end the AIDS epidemic by 2030; 2015. Available at https://www.unaids.org/sites/default/files/media_asset/201506_JC2743_Understanding_FastTrack_en.pdf.

[31] Gonzalez A, Barinas J, O’Cleirigh C. Substance use: Impact on adherence and HIV medical treatment. Curr HIV/AIDS Rep. 2011;8:223. doi: 10.1007/s11904-011-0093-5 21858414

[32] Feldman MB, Thomas JA, Alexy ER, Irvine MK. Crystal methamphetamine use and HIV medical outcomes among HIV-infected men who have sex with men accessing support services in New York. Drug Alcohol Depend. 2015;147:266–271. doi: 10.1016/j.drugalcdep.2014.09.780 25482501

[33] Tomkins A, George R, Kliner M. Sexualised drug taking among men who have sex with men: A systematic review. Perspect Public Health. 2019;139:23–33. doi: 10.1177/1757913918778872 29846139

[34] Hoenigl M, Chaillon A, Moore DJ, Morris SR, Smith DM, Little SJ. Clear links between starting methamphetamine and increasing sexual risk behavior: A cohort study among men who have sex with men. J Acquir Immune Defic Syndr. 2016;71:551–557. doi: 10.1097/QAI.0000000000000888 26536321 PMC4788567

[35] Nerlander LMC, Hoots BE, Bradley H, Broz D, Thorson A, Paz-Bailey G. HIV infection among MSM who inject methamphetamine in 8 US cities. Drug Alcohol Depend. 2018;190:216–223. doi: 10.1016/j.drugalcdep.2018.06.017 30055426 PMC11301976

[36] Rajasingham R, Mimiaga MJ, White J, Pinkston MM, Baden RP, Mitty JA. A systematic review of behavioural and treatment outcome studies among HIV-infected men who have sex with men who abuse crystal methamphetamine. AIDS Patient Care STDS. 2012;26:36–52. 22070609 10.1089/apc.2011.0153 PMC3248609

[37] Colyer SP, Moore DM, Cui Z, Zhu J, Armstrong HL, Taylor M, et al. Crystal methamphetamine use and initiaon among gay, bisexual, and other men who have sex with men living with HIV in a Treatment as Prevention Environment. Subst Use Misuse. 2020;55:2428–2437. 33059493 10.1080/10826084.2020.1833925 PMC7657389

